# Long-Term Control With Proton Beam Therapy for Recurrent Prostate Cancer in the Right Perineum Following Intensity-Modulated Radiation Therapy: A Case Report

**DOI:** 10.7759/cureus.58386

**Published:** 2024-04-16

**Authors:** Yojiro Ishikawa, Motohisa Suzuki, Ichiro Seto, Yoshiaki Takagawa, Masao Murakami

**Affiliations:** 1 Department of Radiology, Tohoku Medical and Pharmaceutical University, Sendai, JPN; 2 Department of Radiation Oncology, Southern Tohoku Proton Therapy Center, Koriyama, JPN; 3 Department of Minimally Invasive Surgical and Medical Oncology, Fukushima Medical University, Koriyama, JPN

**Keywords:** prostate cancer, intensity-modulated radiation therapy (imrt), proton beam therapy, salvage radiotherapy, salvage

## Abstract

Radiation therapy (RT) is commonly used for the treatment of prostate cancer, with intensity-modulated radiation therapy (IMRT) and proton beam therapy (PBT) being the utilized modalities. This case report outlines the treatment course of a recurrent prostate cancer lesion in the right perineal musculature managed with proton therapy following IMRT. A 64-year-old Japanese man, diagnosed with prostate cancer and categorized as high risk according to the National Comprehensive Cancer Network guidelines, underwent six months of androgen deprivation therapy, which included bicalutamide and degarelix acetate. Six months after completing 78 Gy in 39 fractions of IMRT, the patient reported perineal to anal pain. Laboratory tests showed an elevated serum prostate-specific antigen (PSA) level, and pelvic MRI showed a mass lesion in the right perineal musculature. Consequently, the patient was diagnosed with recurrent prostate cancer. Thereafter, the patient underwent eight cycles of systemic chemotherapy with docetaxel; however, his pain progressively worsened. Subsequently, the treatment was switched to 12 cycles of cabazitaxel, which led to gradual pain relief. The patient received PBT at 60 Gy relative biological effectiveness in 30 fractions for the recurrent lesion. Five years after PBT, pelvic MRI showed no mass lesions in the prostate or surrounding tissues. The PSA levels remained low, less than 0.008 ng/ml, and there were no apparent late complications.

## Introduction

External beam radiation therapy (EBRT) for prostate cancer, including intensity-modulated radiation therapy (IMRT) and particle therapy, is used to treat prostate cancer in Japan. The utilization of IMRT and particle therapy, especially proton beam therapy (PBT), is increasing, and there is a growing number of cases of recurrence after EBRT [1,2]. Recently, there have been some reports on salvage therapy options, such as radical prostatectomy (RP), high-intensity focused ultrasound (HIFU), cryotherapy, stereotactic body radiotherapy (SBRT), low-dose-rate (LDR) brachytherapy, and high-dose-rate (HDR) brachytherapy. However, it is challenging to assert that these therapies have been firmly established as treatment strategies [3].

PBT offers a more conformal dose delivery due to its unique characteristic of the Bragg peak, allowing high-dose delivery to the target while reducing exposure to surrounding tissues. There have been numerous reports on the treatment of recurrent malignancies after RT [4,5]. Remarkably, there has been no report to date on salvage PBT for post-RT prostate cancer. This case report describes the long-term results of salvage PBT in a case of local recurrence at the periphery of the irradiated field following initial treatment with IMRT for prostate cancer.

## Case presentation

**Figure 1:**
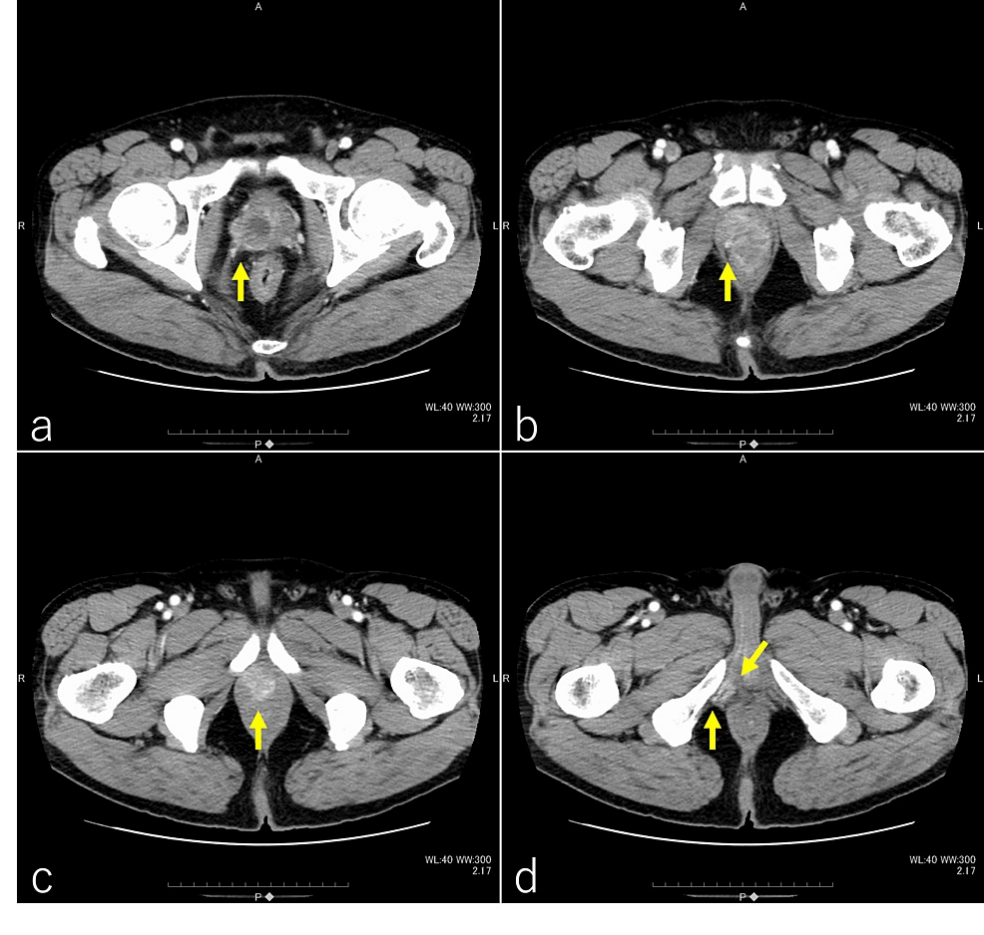
Pelvic contrast-enhanced CT The prostate is predominantly enlarged on the right side, with a visible mass displaying a contrast effect (indicated by arrows in a and b). There is involvement extending toward the apex of the prostate (indicated by an arrow in c) and invasion into the ischiocavernosus muscle and the superficial transverse perineal muscle (indicated by arrows in d).

**Figure 2:**
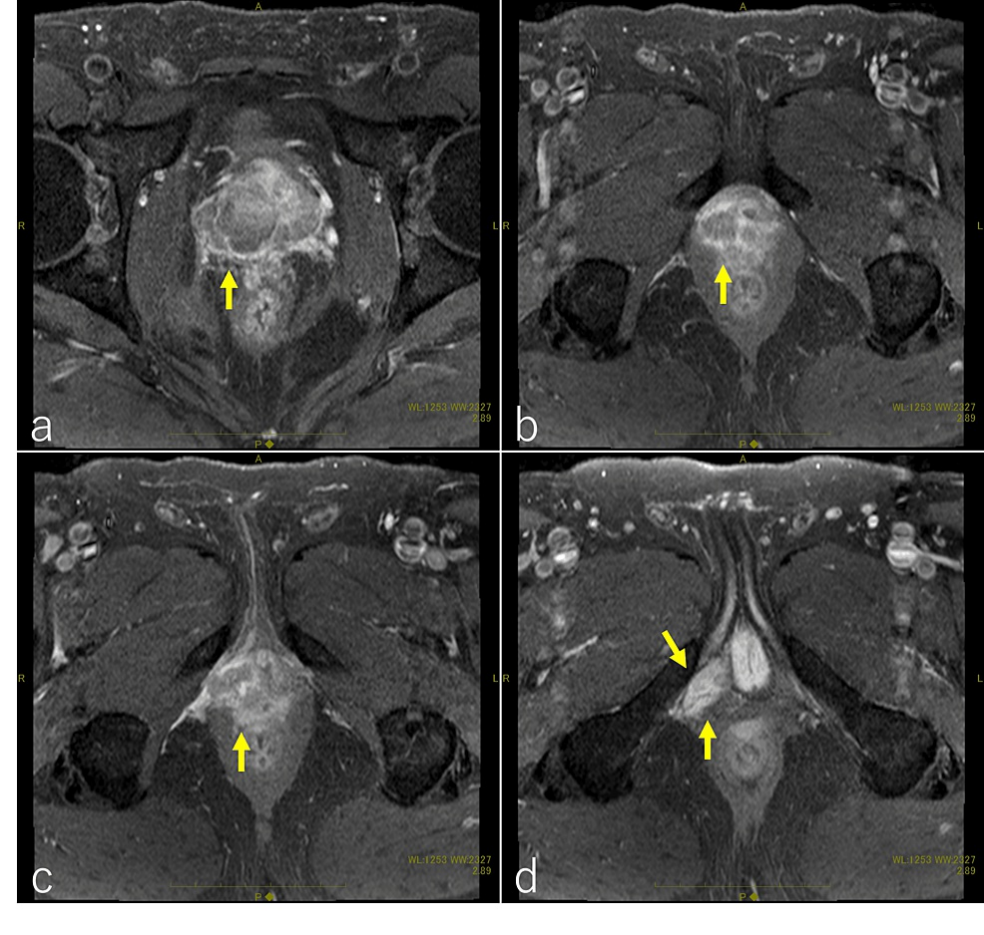
Pelvic contrast-enhanced MRI The MRI reveals a mass lesion in the prostate, consistent with the CT findings. The prostate is primarily enlarged on the right side, and a contrast-enhancing mass is visible (indicated by arrows in a and b). There is infiltration toward the apex of the prostate (indicated by an arrow in c), with suspected involvement of the pubococcygeal and puboanal muscles, along with infiltration of the intraosseous trabecularis muscle and the superficial transverse perineal muscle (indicated by arrows in d).

A 64-year-old Japanese man consulted a gastroenterologist for pain from the perineum to the anus. A CT scan revealed a tumor in the right lobe of the prostate. The tumor extended into the deep perineal space (Figure 1a, 1b, 1c, 1d). The serum test revealed a prostate-specific antigen (PSA) level of 47.834 ng/ml. A prostate biopsy revealed adenocarcinoma with a Gleason score of 4+4. A pelvic MRI scan showed a mass region infiltrating the surrounding tissue (Figure 2a, 2b, 2c, 2d). The clinical stage was diagnosed as T4N0M0 according to the 8th edition of the Union for International Cancer Control.

**Figure 3:**
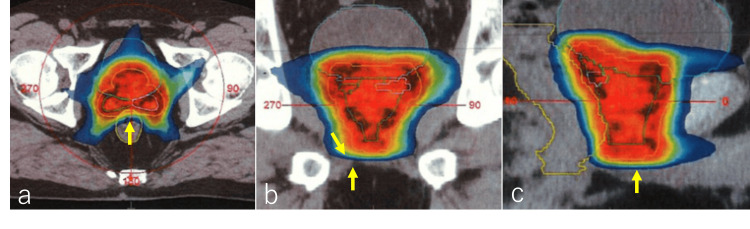
Intensity-modulated radiation therapy Intensity-modulated radiation therapy delivered a total dose of 78 Gy in 39 fractions. Axial (a), coronal (b), and sagittal (c) images are presented, highlighting the tumor-infiltrating lesion in the muscle on the right side of the prostatic apex, as previously identified in CT and MRI scans, which was targeted for irradiation with a submarginal approach (indicated by arrows in b).

The patient was diagnosed with prostate cancer, categorized as a very high-risk group according to the National Comprehensive Cancer Network (NCCN) guidelines, and was administered combined androgen deprivation therapy (ADT) including bicalutamide (80 mg/day) and degarelix acetate (standard dosage of 240 mg in the first month followed by monthly injections of 80 mg) for six months. Laboratory investigations revealed a serum PSA value of 0.116 ng/ml, and pre-treatment images also did not reveal any obvious tumors. The patient received IMRT to a total dose of 78 Gy in 39 fractions delivered over eight weeks for the prostate and seminal vesicles (Figure 3a, 3b, 3c).

**Figure 4:**
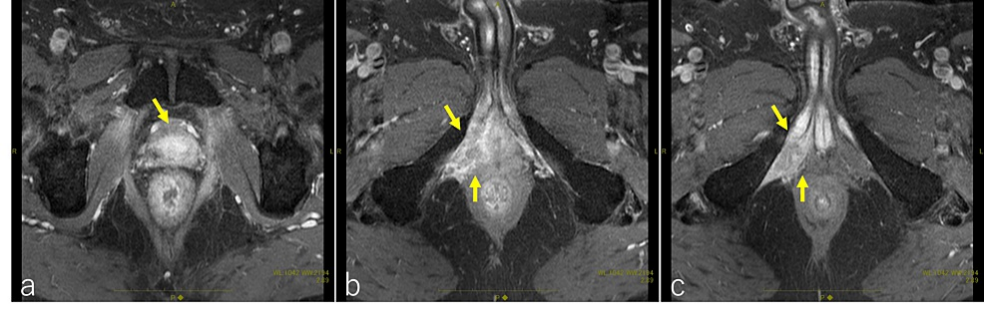
Pelvic contrast-enhanced MR after intensity-modulated radiation therapy A contrast-enhancing recurrent tumor was observed. Although recurrence within the prostate was not evident (indicated by an arrow in a), there was involvement of the intraosseous trabecularis muscle and the superficial transverse perineal muscle (indicated by arrows in b and c).

The patient reported pain extending from the perineum to the anus six months after undergoing IMRT. Subsequent serum testing revealed an elevated serum PSA value (11.99 ng/ml), and a pelvic MRI showed a mass lesion on the right deep perineal pouch (Figure 4a, 4b, 4c).

**Figure 5:**
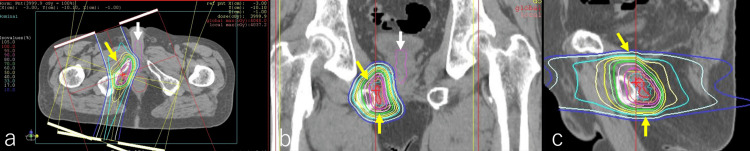
Dose distribution of proton beam therapy Proton beam therapy (PBT) planning and delivery techniques were employed, and the patient underwent passive-scattering proton therapy using a PBT system from Mitsubishi Electric in Kobe, Japan. Treatment planning was performed using CT simulation with an Aquilion LB machine from Toshiba Medical Systems in Tokyo, Japan, set to a 2.0 mm slice thickness. The prescribed dose was 60 Gy relative biological effectiveness (RBE), covering 95% of the PTV in 2 Gy (RBE) fractions. The PBT dose distribution is shown by the yellow arrow in the axial (a), coronal (b), and sagittal (c) images. Treatment planning was optimized according to our institutional constraints: for the rectum, the volume of the rectum receiving at least 40% of the prescribed radiation dose (V40) < 35% and V65 < 17%, and for the bladder, the volume of the bladder receiving at least 40% of the prescribed radiation dose (V40) < 50% and V65 < 25% (indicated by the yellow arrow). The dose overlap with the previous irradiation for the urethra was adjusted to ensure it did not exceed approximately 90 Gy (indicated by the white arrow).

**Figure 6:**
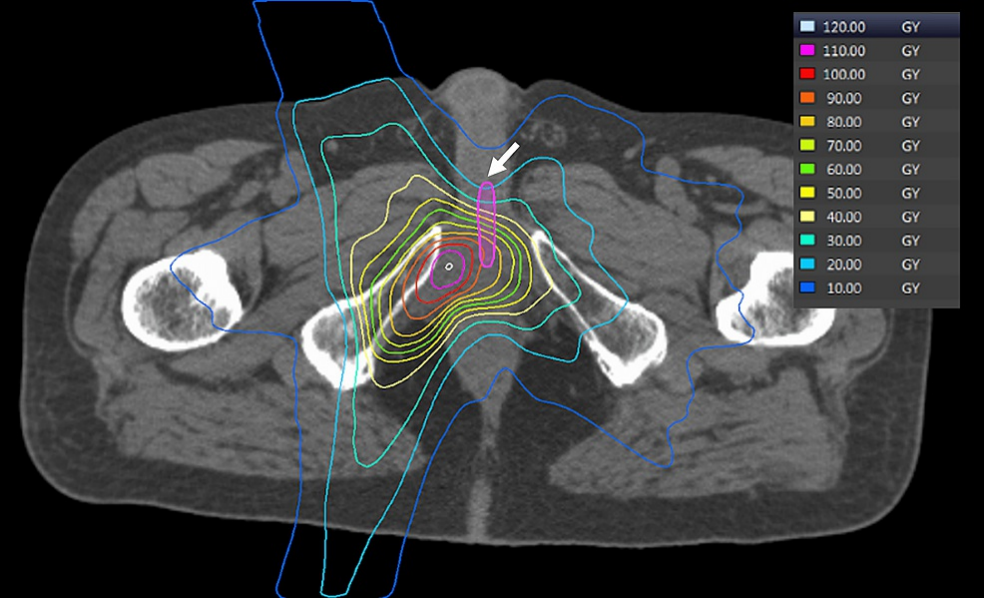
Distribution of the cumulative doses from the initial intensity-modulated therapy and proton beam therapy The combined dose distribution, including the initial intensity-modulated therapy delivering 78 Gy and the irradiation from the 60 Gy relative biological effectiveness proton beam therapy, is demonstrated. The maximum dose reached 120 Gy at the tumor site, while the dose to the urethra was kept below 90 Gy (indicated by the arrow).

The patient was diagnosed with a recurrence of prostate cancer. ADT with degarelix acetate proved ineffective in treating the recurrent lesion. Consequently, the patient underwent systemic chemotherapy with docetaxel (75 mg/m^2^) for eight cycles; however, his pain gradually increased, and the PSA value did not decrease. As a result, the patient's chemotherapy regimen was changed to cabazitaxel (25 mg/m^2^) for 12 cycles. After switching to this chemotherapy regimen, the pain gradually subsided. Serum PSA levels decreased to 0.140 ng/ml. The patient experienced side effects from the chemotherapy, specifically developing hematologic toxicity and numbness equivalent to Grade 3 according to the Common Terminology Criteria for Adverse Events, and sought consultation regarding alternative therapies for PBT. The patient received passive-scattering PBT at 60 Gy relative biological effectiveness (RBE) in 30 fractions for the recurrent lesion at our institution (Figure 5a, 5b, 5c). The prescribed dose was 60 Gy RBE, covering 95% of the PTV in 2 Gy (RBE) fractions. The gross tumor volume (GTV) was defined as the recurrent tumor in the right deep perineal pouch. The clinical target volume (CTV) was subsequently defined as the GTV plus an additional 0.5 cm margin. The planning target volume (PTV) was subsequently defined as the CTV plus an additional 0.5 cm margin. The dose overlap with the previous irradiation for the urethra was adjusted to ensure it did not exceed approximately 90 Gy. We administered a luteinizing hormone-releasing hormone (LH-RH) agonist (leuprorelin acetate) after PBT (Figure 6).

**Figure 7:**
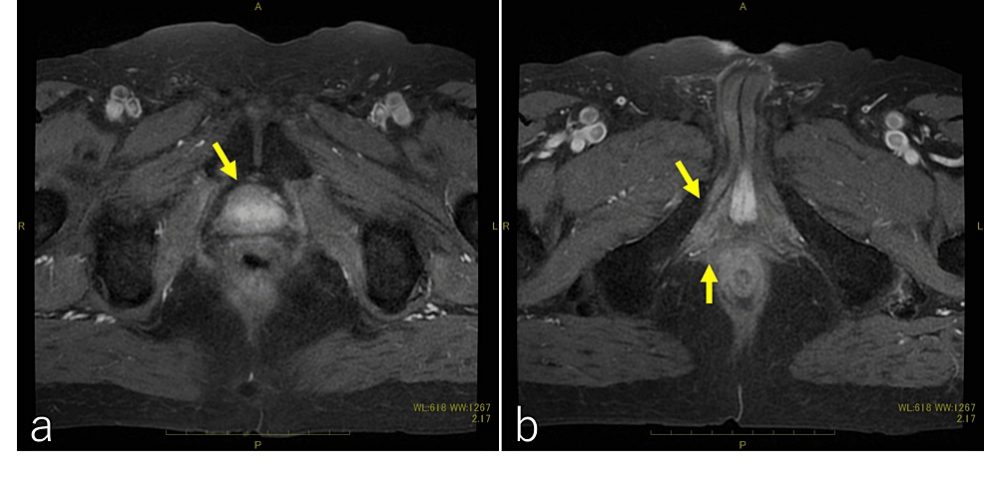
Pelvic contrast-enhanced MRI after proton beam therapy A contrast-enhancing recurrent tumor was not observed after proton beam therapy (indicated by an arrow in a); there was also no recurrence of the tumor in the intraosseous trabecularis muscle and the superficial transverse perineal muscle (indicated by arrows in b).

Five years post-PBT, the patient remained in complete remission with the LH-RH agonist alone. There were no complications after PBT. Laboratory investigations revealed a serum PSA value of less than 0.008 ng/ml. Pelvic MRI revealed no mass lesion in the prostate or surrounding tissue (Figure 7a, 7b).

## Discussion

The outcomes of IMRT for prostate cancer are comparable to those of surgery with a five-year rate of biochemical relapse-free survival (bRFS) of approximately 90% [6-9]. There have been several reports on the results of salvage therapy in surgery, with an approximate control rate of 30-50%. Post-irradiation surgery is not a preferred treatment option due to complications, such as suture failure, infection, and urine leakage [10]. On the other hand, brachytherapy can be performed with relatively few side effects, and there have been some reports of its use [11]. There have also been a few reports of high-intensity focused ultrasound (HIFU) treatment being effective [12]. In the present case, local recurrence at the periphery of the irradiated field following initial treatment with IMRT for prostate cancer extended beyond the prostate into the surrounding musculature. Typically, brachytherapy, HIFU, and surgery have not demonstrated effectiveness for recurrence that extends into the musculature beyond the prostate [10-12].

In this case, PBT was administered at a dose of 60 Gy (RBE), which was relatively low for radical prostate cancer treatment. The decision to use a lower re-irradiation dose of 60 Gy was made to protect the surrounding organs. It was challenging to believe that the 60 Gy dose alone controlled the recurrent lesions, as radical irradiation for prostate cancer typically involved doses of at least 70 Gy [1-3,5]. Chemotherapy might have contributed to favorable local control in this instance. While docetaxel did not demonstrate significant effectiveness, cabazitaxel showed effectiveness in alleviating symptoms and reducing PSA levels in this case. Although chemotherapy has been established as effective in castration-resistant prostate cancer [13,14], it is noteworthy that, in this case, the addition of PBT rendered additional chemotherapy unnecessary. It is known that recurrence near the apex of the prostate is common [15]. It was previously reported that 69 of 77 patients (90%) who underwent salvage radical prostatectomy and did not respond to initial RT had recurrence tumors at the apex. The same report also mentioned that many recurrence tumors also occur in the distal apex, periurethra, and seminal vesicles [16]. The present case is considered exceptionally rare as the lesion extended from the apex of the prostate into the surrounding musculature.

Since IMRT is increasingly becoming the treatment of choice for prostate cancer, there has been an increase in recurrences after IMRT [8,9]. The salvage option after local recurrence is radical prostatectomy, if pursued with curative intent. Androgen deprivation therapy (ADT) is generally one of the preferred salvage therapies for biochemical recurrence after RT. However, it is important to note that prostate cancer is not completely cured by ADT, and eventually becomes resistant to it. Androgen-dependent cancers lose their androgen dependence and continue to grow, exhibiting castration resistance within two to three years [17]. ADT is often continued for a long period of time in many recurrence cases after RT for prostate cancer. Although ADT is very effective and useful for recurrent prostate cancer after RT [18], there are problems that lead to physical and psychological stress for patients, such as financial stress from long-term administration of expensive hormone preparations and side effects of ADT (hot flashes, osteoporosis, depression, etc.) [18,19]. The patient has been on ADT for five years, with no apparent adverse reactions observed.

One limitation to consider is that this prolonged usage may have influenced the treatment's overall outcome. In this case, the prostate cancer was confined to the peri-prostatic region, and there were no distant metastases, which may have had a favorable influence on the outcome. Although there was an overlap between IMRT and proton therapy in this case, the musculoskeletal muscles can tolerate high doses to a certain extent, and the possibility of re-irradiation with proton therapy was demonstrated if attention is paid to doses to the intestinal tract, urinary tract, and skin. It should be noted, however, that this is only a case report and does not suggest a standard treatment for recurrence after IMRT for prostate cancer.

## Conclusions

This case report describes the long-term results of salvage PBT in a case of local recurrence at the periphery of the irradiated field following initial treatment with IMRT for prostate cancer.

While it is essential to acknowledge that this report does not establish a standard treatment protocol for recurrence after IMRT for prostate cancer, it holds significance for patients meeting the criteria of responding to chemotherapy and being eligible for a curative dose of approximately 60 Gy. For patients without distant metastases, careful assessment of radiation dosage to critical organs, such as the rectum, urethra, bladder, and skin, is advisable when considering salvage therapy. Salvage therapy may be considered as an option for cases without distant metastases, with attention to the dosages administered to these vital organs.
